# Bilateral Internal Carotid Artery Agenesis in a Patient With a Family History of Intracranial Pathology

**DOI:** 10.31486/toj.22.0052

**Published:** 2023

**Authors:** Yi-Ming J. Liau, Austin J. Jabbour, Heather Yerdon, Carlos Cevallos Chonillo, Saira Amjed, Andrew Hong, Behram Khan

**Affiliations:** ^1^ The University of Queensland Medical School, Ochsner Clinical School, New Orleans, LA; ^2^ Institute of Translational Research, Ochsner Clinic Foundation, New Orleans, LA; ^3^ Department of Neurology, Ochsner Clinic Foundation, New Orleans, LA; ^4^ Department of Internal Medicine, Ochsner Clinic Foundation, New Orleans, LA; ^5^ Department of Hospital Medicine, Ochsner Clinic Foundation, New Orleans, LA

**Keywords:** *Carotid artery diseases*, *intracranial arterial diseases*, *neuroanatomy*, *vertebrobasilar insufficiency*

## Abstract

**Background:** Agenesis of the internal carotid artery (ICA) is a rare congenital malformation that is often asymptomatic until the fourth or fifth decade. ICA agenesis is associated with several intracranial pathologies, the most reported being intracranial aneurysms, thought to be attributable to the increased flow in the collateral vessels supplying the anterior circulation. The cause of ICA agenesis is largely unknown and has not been consistently associated with any genetic mutations or syndromes.

**Case Report:** We present the case of a 37-year-old female who was incidentally found to have bilateral agenesis of the ICA system. Patient history revealed that the patient's father and 12 of his 14 siblings died from either ruptured brain aneurysms or cerebrovascular accidents before the age of 50 years. Presenting symptoms included right eye pain radiating to her right posterior neck, a 2-month history of diplopia, and associated nausea and vomiting. Differential diagnoses included immunoglobulin G4–related disease, sarcoidosis, lymphoma, and vasculitis. Absent internal carotids were attributed to congenital agenesis vs hypoplasia. The patient was seen by neurology and initiated on prednisone 80 mg by mouth once daily with a 2-week taper to treat systemic inflammation. The patient was deemed stable for discharge after a 2-day hospital admission and was scheduled for follow-up appointments with genetics, neurology, rheumatology, and ophthalmology.

**Conclusion:** Bilateral ICA agenesis is a rare occurrence, with only 33 cases documented in a case report and literature review published in 2016. Because of the otherwise normal anatomy of the patient and the pervasive intracranial pathology seen in late adulthood in her family, we propose the likelihood of an inheritable form of bilateral ICA agenesis vs vascular disease or familial aneurysms.

## INTRODUCTION

Agenesis of the internal carotid artery (ICA) is a very rare event, occurring in 0.01% of the population. Bilateral cases are even rarer. In a case report and literature review published in 2016, Alexandre et al reported a total of 33 documented cases of bilateral agenesis of the ICA.^1^ The cause of ICA agenesis, unilateral and bilateral, is largely unknown and has not been linked with any genetic abnormalities. ICA agenesis has been observed conjointly with rare genetic disorders, but these reports have been anecdotal, with no hypotheses proposed to explain the association.^2-4^

We describe a case of bilateral agenesis of the ICA system in a class III obese African American adult who had no known genetic disorders.

## CASE REPORT

A 37-year-old African American female with a body mass index of 62 kg/m^2^ and a history of hypertension, type 2 diabetes, and alcohol abuse presented to the emergency department (ED) for evaluation of sudden onset, throbbing, right eye pain radiating to her right posterior neck and a 2-month history of diplopia. Full review of systems revealed associated nausea and vomiting. Family history revealed the patient's father and 12 of her father's 14 siblings died from either ruptured brain aneurysms or cerebrovascular accidents before the age of 50 years. Vital signs were remarkable for a blood pressure of 156/92 mm Hg. Examination of the right eye revealed a complete deficit in abduction, partial deficit in upward gaze, and partial deficit in adduction, suggestive of a cranial nerve VI palsy with partial, pupil-sparing cranial nerve III palsy.

**Figure 1.**
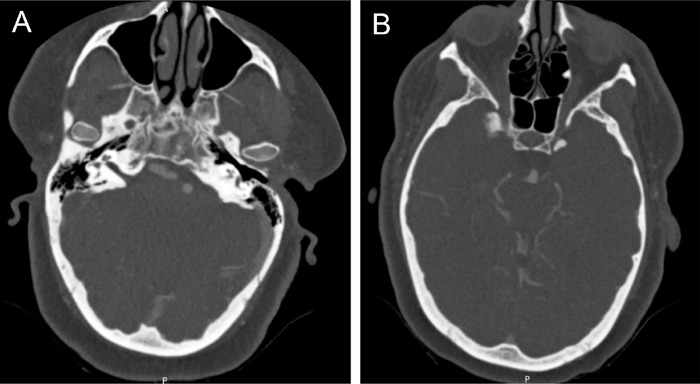
(A) Axial computed tomography angiography (CTA) image of the skull base shows the absence of petrous carotid canals. (B) Axial CTA image of the brain demonstrates absent intracranial carotid flow above the cavernous sinuses.

**Figure 2.**
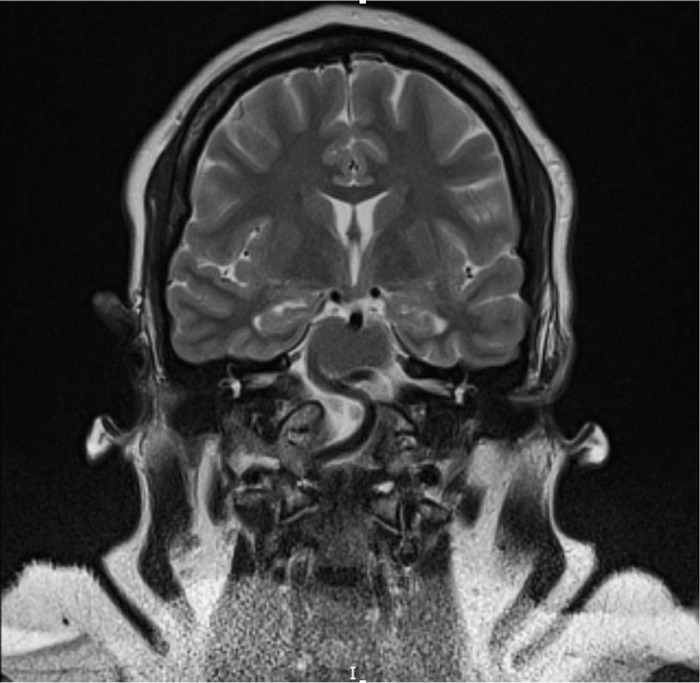
T2 weighted magnetic resonance imaging with contrast shows absent internal carotid artery flow voids through the cavernous segments (apparent absence of the bilateral internal carotid arteries). A tortuous and enlarged basilar artery passes adjacent to the origin of the sixth cranial nerve in the pons.

Computed tomography angiography (CTA) and magnetic resonance imaging (MRI) of the brain were performed to rule out vascular etiologies. Notable CTA incidental findings included the absence of the bilateral carotid canals, intracranial internal carotid arteries, and cavernous sinuses (Figure 1). MRI revealed vertebrobasilar dolichoectasia that was notably adjacent to the origin of the sixth cranial nerve in the region of the pons with resultant compression (Figure 2). Ill-defined enhancement surrounded the right optic nerve with resultant asymmetric proptosis, thought to be idiopathic orbital inflammation. Brain parenchyma was normal, and no hydrocephalus or aneurysms were identified. Computed tomography (CT) of the chest, abdomen, and pelvis was unremarkable for acute pathology.

Complete blood count and comprehensive metabolic panel were grossly unremarkable. Inflammatory markers were significantly elevated, with erythrocyte sedimentation rate of 96 mm/h (reference range, 0-36 mm/h) and C-reactive protein of 59.2 mg/L (reference range, 0-8.2 mg/L). Rheumatologic testing revealed negative antinuclear antibody and elevated C3 at 199 mg/dL (reference range, 50-180 mg/dL). Cerebrospinal fluid analysis was significant for an elevated opening pressure in the seated position at 36 mm Hg (reference range, 5.1-13.2 mm Hg in the lateral recumbent position).

Differential diagnoses included immunoglobulin G4–related disease, sarcoidosis, lymphoma, and vasculitis. Absent internal carotids were attributed to congenital agenesis vs hypoplasia. The patient was seen by neurology and initiated on prednisone 80 mg by mouth once daily with a 2-week taper to treat systemic inflammation. The patient was deemed stable for discharge after a 2-day hospital admission and was scheduled to receive close follow-up appointments with genetics, neurology, rheumatology, and ophthalmology.

The patient presented to the genetics department 3 weeks later for workup of potential heritable causes of her neurovascular abnormalities. However, her visit ended prematurely because of vomiting and shortness of breath, for which she was taken to the ED. She was found to be dehydrated secondary to decreased oral intake and increased alcohol use because of the recent death of her mother. She was given fluids and discharged after 1 day in observation.

At the time of this report, the patient had not scheduled another appointment with genetics and had not followed up with neurology or rheumatology. However, she did report for the ophthalmology appointment 2 months later, and her eye-related symptoms had resolved.

## DISCUSSION

Agenesis, hypoplasia, and aplasia of the ICA are often used interchangeably in the literature. However, ICA agenesis is defined as the complete absence of the vessel and branches. It is differentiated from aplasia or hypoplasia by the absence of all segments and by an underdeveloped carotid canal on imaging.^5^ In a fetus, the ICA is formed 2 weeks before the skull base and is thus required as a scaffold for proper skull base development. Agenesis of the ICA is thought to take place during the third and fifth week of embryologic development during simultaneous regression of the first and third aortic arches.^6^ Imaging in this patient revealed the absence of the bilateral ICAs and carotid canals, thus supporting ICA agenesis vs postbirth hypoplasia, aplasia, or occlusion.

ICA agenesis, aplasia, and hypoplasia have been reported to be associated with numerous intracranial pathologies that appear in later years such as headaches, seizures, tinnitus, strokes, transient ischemic attacks, and Horner syndrome.^7^ However, the most prevalent pathology associated with ICA agenesis in the literature has been intracranial aneurysms and consequent subarachnoid hemorrhages^8^ resulting from increased volume and abnormal flow dynamics in the collateral vasculature caused by deficient anterior circulation.^9^ Over time, the vessels dilate and become torturous, as seen in the vertebrobasilar circulation in our patient, and eventually lead to weakening of the vessel walls and aneurysms. Therefore, many patients are asymptomatic until the fourth or fifth decade.^10^ Zink et al reported a prevalence of intracranial aneurysm in patients with congenital hypoplasia or aplasia of the ICA in up to 14.6% in patients <30 years of age and 36.6% in patients >30 years.^10^ According to Zink et al, the increased prevalence of aneurysms in the older group suggests that the pathogenesis of the aneurysms in their study population was more likely secondary to chronic stress on the vessels rather than intrinsic vascular disease.

Nevertheless, some authors have proposed that ICA agenesis is merely a symptom of a congenital vascular disease.^11^ Several well-known hereditary conditions have been known to affect the vasculature and cause intracranial aneurysms. Polycystic kidney disease (PKD), caused by alterations in the PKD1/2 gene, is associated with berry aneurysms and vertebral dissection. Ehlers-Danlos syndrome (EDS) can lead to cerebral aneurysm secondary to a COL3A1 mutation.^11^ Other conditions that can affect the vasculature and cause cerebral aneurysms include neurofibromatosis type 1 (NF1), multiple endocrine neoplasia type 1 (MEN1), and hereditary hemorrhagic telangiectasia (HHT).^12^

These inherited conditions are unlikely in our patient, as her CT scans of the chest, abdomen, and pelvis were without typical findings of thoracic artery aneurysm or bilateral polycystic kidneys. She had no signs of telangiectasias or of neurofibromas. The patient also lacked findings characteristic of EDS, such as a marfanoid body habitus or hyperextensibility of joints and ligaments. In addition, cerebral aneurysms occur in a small percentage of patients with the above-mentioned genetic diseases. Therefore, given the fact that 12 of the patient's father's 14 siblings died from brain complications, these familial disorders are not likely to have caused the high prevalence of aneurysms seen in the patient's family.

Many cases of familial occurrence of intracranial aneurysms have been reported without a known heritable condition.^12^ Familial aneurysms are largely idiopathic, with reported mutations spanning many different chromosomes and loci, and in addition, having equally variable inheritance patterns.^12,13^ The patient's family possibly could have had a severe form of familial aneurysms in the absence of a known genetic mutation; however, aneurysms tend to occur at younger ages, in multiples, and with a predilection for the middle cerebral artery.^12-14^ Our patient had no visible aneurysms on CT or MRI.

Based on our patient's imaging and history, we believe the intracranial aneurysms and cerebrovascular pathology in her family were likely attributable to hereditary ICA agenesis and consequent high-volume flow in the collateral circulation. Our patient did not have physical examination findings consistent with the genetic diseases that cause intracranial aneurysms (PKD, EDS, NF1, MEN1, HHT). Also, unlike patients with familial aneurysms, who often have multiple aneurysms at a young age, our patient's imaging was grossly normal other than the ICA agenesis and vertebrobasilar dolichoectasia. In addition, while the incidence of familial aneurysms can be as high as 19.1% in siblings,^12^ the prevalence of aneurysms with single ICA agenesis is estimated to be much higher at 36.6%.^10^ In fact, if the mechanism of the aneurysms is chronically stressed collaterals as we propose, bilateral agenesis could yield an even higher prevalence of aneurysms than unilateral agenesis and explain the permeating intracranial pathology in the patient's family.

Without a detailed genetic workup of the patient and her family and only subjective reporting of the patient's family history, we acknowledge that the conclusions we can make are limited. The patient also had significantly elevated inflammatory markers; therefore, a rheumatology workup would be needed to rule out autoimmune or inflammatory disease. However, if the cause of the patient's family history is inherited ICA agenesis, to our knowledge, this case is the first report of such a phenomenon.

## CONCLUSION

While ICA agenesis can be asymptomatic for most of adulthood, it should be taken seriously as a strong risk factor for cerebral aneurysms and other cerebrovascular accidents. When ICA agenesis is found incidentally, as most cases are, physicians should understand the importance of surveillance and identifying collateral pathways supplying the brain that may eventually succumb to disease. The literature offers few explanations for patients with no obvious genetic abnormalities and a strong familial history of brain aneurysms. For these patients, abnormalities in the main vessels supplying their brain should be investigated.

## Notes

*This article meets the Accreditation Council for Graduate Medical Education and the American Board of Medical Specialties Maintenance of Certification competencies for Patient Care and Medical Knowledge.*
